# Can vagus nerve stimulation halt or ameliorate rheumatoid arthritis and lupus?

**DOI:** 10.1186/1476-511X-10-19

**Published:** 2011-01-24

**Authors:** Undurti N Das

**Affiliations:** 1 UND Life Sciences, 13800 Fairhill Road, #321, Shaker Heights, OH 44120, USA; 2 School of Biotechnology, Jawaharlal Nehru Technological University, Kakinada-533 003, India

## Abstract

Acetylcholine, the principal vagus neurotransmitter, inhibits inflammation by suppressing the production of pro-inflammatory cytokines through a mechanism dependent on the α7 nicotinic acetylcholine receptor subunit (alpha7nAChR) that explains why vagus nerve stimulation is anti-inflammatory in nature. Strong expression of alpha7nAChR in the synovium of rheumatoid arthritis and psoriatic arthritis patients was detected. Peripheral macrophages and synovial fibroblasts respond in vitro to specific alpha7nAChR cholinergic stimulation with potent inhibition of proinflammatory cytokines. Fibroblasts balance inflammatory mechanisms and arthritis development through feedback cholinergic stimulation by nearby immune cells. Collagen induced arthritis in alpha7nAChR^(-/-) ^mice was significantly severe and showed increased synovial inflammation and joint destruction compared to the wild-type mice. Similar to vagal nerve stimulation and alpha7nAChR agonists, polyunsaturated fatty acids: eicosapentaenoic acid (EPA) and docosahexaenoic acid (DHA) also suppress inflammation. In view of their similar anti-inflammatory actions, it is proposed that vagal nerve stimulation, alpha7nAChR agonists and EPA and DHA may augment the formation of anti-inflammatory lipid molecules: lipoxins, resolvins, protectins and maresins. This implies that therapies directed at regulation of the cholinergic and alpha7nAChR mediated mechanisms and enhancing the formation of lipoxins, resolvins, protectins and maresins may halt and/or ameliorate rheumatoid arthritis, lupus and other rheumatological conditions.

## Introduction

Rheumatoid arthritis (RA) is a chronic, inflammatory autoimmune disease of unknown cause, characterized by chronic synovial inflammation and progressive erosions of cartilage and bone due to the release of excess cytokines that stimulate the inflammatory reaction. Systemic lupus erythematosus or lupus is also a chronic inflammatory autoimmune connective tissue disease that can affect any part of the body resulting in inflammation and tissue damage that often targets the heart, joints, skin, lungs, blood vessels, liver, kidneys and nervous system. Both RA and lupus show several overlapping features though are considered clinically distinct entities. Inflammation plays a major role in these and other autoimmune diseases that involve a complex interaction between several pro-inflammatory cytokines as well as chemokines, neurotransmitters and other signalling molecules.

Inflammation is at the core of the autoimmune diseases that seem to arise through aberrant reactions of the human adaptive or innate immune systems due to the activation of the patient's immune system against the body's own proteins. As a result, a series of pro-inflammatory molecules such as tumor necrosis factor-α (TNF-α), interleukin-6 (IL-6), macrophage migration inhibitory factor (MIF), interferon (IFN), high-mobility group box-1 (HMGB-1), free radicals, myeloperoxidase and inducible nitric oxide (iNO) are released that initiate and perpetuate the inflammatory process. Though the exact cause for autoimmune disorders is not known, it is clear that methods designed to suppress the aberrant inflammatory response are of significant help in giving relief to the patients. For this purpose, non-steroidal anti-inflammatory compounds, corticosteroids, anti-TNF-α, and immunosuppressive drugs are widely used in the management of rheumatoid arthritis (RA) and lupus and other autoimmune diseases [1]. Despite the use of these drugs, majority of the patients continue to suffer from the various manifestations of these diseases and relatively few attain complete remission. Furthermore, not all patients respond adequately to the various drugs used in their treatment that are not without significant side-effects. In view of this, it is imperative that newer approaches are needed to manage RA, lupus and other related conditions.

Recent studies suggested that cholinergic mechanisms regulate immune function. T and B cells and macrophages have the ability to produce acetylcholine (ACh) and enzymes involved in ACh production, i.e. choline acetyl transferase (ChAT) [2-4]. These cell types also express various cholinergic receptors on their surface. Of all, nicotinic receptors with the subtype a7 (alpha7nAChR) have significant effects on immune regulation [4]. Specific stimulation of alpha7nAChR on monocytes suppresses pro-inflammatory cytokine production termed as the cholinergic antiinflammatory pathway [5-8]. These evidences suggest that cholinergic antiinflammatory pathway could be exploited to suppress inflammation seen in RA, lupus and other autoimmune diseases.

## Synovial tissue express alpha7nAChR

Recent studies revealed that alpha7nAChR is present in the synovial tissue of healthy volunteers. In the synovial sublining, alpha7nAChR was found to be expressed in scattered cells with macrophage- and fibroblast-like morphology. Strong staining of alpha7nAChR was detected in perivascular endothelial-like cells with visible vessels. Alpha7nAChR is detected in synovial tissue of all RA and psoriatic arthritis patients and is especially present in the synovial lining layer than the sublining. In patients with follicular mononuclear infiltrates, the alpha7nAChR expression was mainly observed in cells with macrophage like morphology. Furthermore, macrophages (expressing CD68 and CD163) were found to strongly express alpha7nAChR as well as fibroblasts, in occasional T cells (CD3) and B cells (CD19) including plasma cells (CD138) and CD19 positive cells in a mononuclear infiltrate [9]. These results suggest that specific expression of cholinergic receptors is seen in the synovium of normal and those with RA and psoriasis. In addition, several cell types in the arthritis synovium are capable of cholinergic stimulation. Based on these studies, it was opined that the diversity in cholinergic immune regulation may contribute to the different pathological and clinical features of RA and psoriatic arthritis. The presence of alpha7nAChR in macrophages, T cells, B cells and plasma cells suggest that specific cholinergic mechanisms may be involved in regulation of antibody production locally in the joint. Cholinergic mechanisms may also influence synovial production of autoantibodies that are known to be involved in RA pathogenesis. The presence of alpha7nAChR receptors on endothelial cells might contribute to the regulation of endothelial cell proliferation and migration [10,11] and angiogenesis [12] and thus, cholinergic mechanisms may have an impact in the pathobiology of arthritis.

## Cholinergic anti-inflammatory pathway

Central nervous system is known to have a regulatory role in the production of pro-inflammatory cytokines: tumor necrosis factor-α (TNF-α), interleukin-1 (IL-1), high mobility group box 1 (HMGB1), IL-6, and macrophage migration inhibitory factor (MIF) through the efferent vagus nerve [13-15]. Acetylcholine, the principal vagus neurotransmitter, inhibits the production of pro-inflammatory cytokines through a mechanism dependent on the α7 nicotinic acetylcholine receptor subunit (alpha7nAChR). Thus, vagus nerve stimulation controls the production of pro-inflammatory cytokines. Since strong expression of alpha7nAChR is seen in the synovium of normal, RA and psoriatic arthritis patients [9], and both peripheral macrophages and synovial fibroblasts respond to specific alpha7nAChR cholinergic stimulation with potent inhibition of proinflammatory cytokines [13-15], it is likely that fibroblasts, especially in the lining layer, balance inflammatory mechanisms and arthritis development through feedback cholinergic stimulation by nearby immune cells. These evidences suggest that new therapies directed at regulation of the cholinergic and alpha7nAChR mediated mechanisms could be of benefit in the management of lupus, RA and other related inflammatory and immune disorders.

## Cholinergic anti-inflammatory pathway is depressed in RA

This proposal is supported by the observation that RR interval variability (heart rate variability, HRV) that is a marker of vagus nerve tone (that reflects parasympathetic activity), in RA patients was significantly depressed compared to control [16]. These findings imply that it is possible to pharmacologically target the alpha7nAChR dependent control of cytokine release in RA patients who have suppressed vagus nerve activity. Alpha7nAChR agonists ameliorated the clinical course of collagen induced arthritis in animals [17]: arthritis was exacerbated by vagotomy and suppressed by oral nicotine administration; oral nicotine inhibited bone degradation and reduced TNF-α expression in synovial tissue, and ameliorated clinical arthritis and reduced synovial inflammation that was accompanied by a reduction of TNF-α level in both plasma and synovial tissue. Histopathological studies showed that nicotine reduced infiltration of inflammatory cell and bone destruction, and decreased expression of TNF-α and IL-6 but did not alter IL-10 levels. Nicotine also reduced the expression and translocation of HMGB1 in the inflamed joints of collagen-induced arthritis mice [18].

Furthermore, RR interval variability in RA patients was significantly decreased as compared with controls. HMGB1 levels and RR interval variability were significantly related. HMGB1 serum levels significantly correlated with disease activity scores in patients with RA. These results are consistent with the hypothesis that decreased cholinergic anti-inflammatory pathway activity is associated with increased HMGB1 levels in patients with RA [19].

It was reported that RR interval variability, in RA patients was significantly depressed compared to normal control. In the same group of RA patients, whole blood cultures stimulated by endotoxin produced significantly less TNF as compared to healthy controls. Addition of cholinergic agonists to the stimulated whole blood cultures however significantly suppressed cytokine production to a similar extent in patients and healthy controls [16]. In addition, nicotine reduced the protein and mRNA expression of IL-6 and IL-8 induced by TNF-α, inhibited nuclear factor (NF)-kappaB (p65) translocation from the cytoplasm to the nucleus [20,21]. These results suggest that alpha7nAChR agonists modulate the release of pro-inflammatory cytokines and thus, are of significant benefit in RA.

Furthermore, collagen-induced arthritis in alpha7nAChR^(-/-) ^mice was significantly severe and showed increased synovial inflammation and joint destruction compared to the wild-type mice. Exacerbation of the arthritis was associated with elevated systemic proinflammatory cytokines and enhanced T-helper cell 1 (Th1)-cytokine and TNF-α production by spleen cells [22]. These results suggest that immune cell function in a model of rheumatoid arthritis is regulated by the cholinergic system and, at least in part, mediated by the alpha7nAChR206. These results imply that vagus nerve stimulation could be employed in the treatment of lupus, RA and other autoimmune and inflammatory diseases.

## Interaction(s) between acetylcholine and polyunsaturated fatty acids

Exogenously added and endogenously produced arachidonic acid (AA) increased the evoked release of acetylcholine from rat hippocampal nerve terminals [23] and possibly, other tissues. Muscarinic activation triggers AA production. AA may act as a facilitatory retrograde messenger in hippocampal cholinergic muscarinic transmission in view of the ability of AA to enhance acetylcholine release. In a similar fashion, dietary docosahexaenoic acid (DHA) enhanced potassium chloride-evoked release of acetylcholine in rat hippocampus [24], suggesting that acetylcholine release is regulated by polyunsaturated fatty acids (PUFAs) [1,25-28]. Acetylcholine and PUFA-derived lipoxins, resolvins, protectins and maresins have anti-inflammatory actions. But it is not known whether acetylcholine can actually enhance the formation anti-inflammatory lipoxins, resolvins, protectins and maresins. In all probability it does.

## Acetylcholine and PUFA metabolism

Torpedo electric organ readily synthesizes prostaglandin E_2 _(PGE_2_) from both exogenous and endogenous arachidonate and activation of the presynaptic muscarinic acetylcholine receptor increases the rate of PGE_2 _synthesis by inducing the release of tissue AA from its phospholipid pools. Torpedo electric organ slices also synthesize PGE_2 _from endogenous substrates. Activation of the Torpedo muscarinic acetylcholine receptor resulted in a dose-dependent increase in the synthesis of PGE_2 _from endogenous AA. These results suggest that activation of the muscarinic acetylcholine receptor enhances the formation of PGE_2 _[29].

Acetylcholine significantly increased the efflux of PGE and PGF from brain regions enriched in muscarinic cholinergic receptors, i.e., cerebral cortex, temporal cortex, corpus striatum, and hippocampus that was inhibited by atropine, while norepinephrine, histamine, and serotonin were ineffective in stimulating PG release from brain cortex slices [30]. Acetylcholine augments the formation of prostacyclin (PGI_2_), a potent vasodilator and anti-inflammatory molecule [31,32]. These evidences suggest that acetylcholine modulates PUFA metabolism and enhances the formation of PGE_2 _and PGI_2_. Hence, it is likely that acetylcholine may augment the formation of lipoxins, resolvins, protectins and maresins. This is especially so since, acetylcholine and lipoxins, resolvins and protectins have anti-inflammatory actions. Thus, acetylcholine and PUFAs may serve as endogenous anti-inflammatory molecules. Similar to the ability of acetylcholine and VNS that ameliorate insulin resistance, even PUFAs reduce insulin resistance [33-35]. This implies that one potential mechanism by which acetylcholine produces its anti-inflammatory action is by enhancing the formation of lipoxins, resolvins, protectins and maresins from PUFAs. Thus, it appears that PUFAs augment release of acetylcholine, while acetylcholine enhances the conversion of PUFAs to anti-inflammatory lipoxins, resolvins, protectins and maresins. Such an interaction between acetylcholine and PUFAs may explain their anti-inflammatory nature and possible beneficial action of VNS in inflammatory conditions such as RA and lupus [17-22,36-40].

## Hypothesis

**Figure 1:**
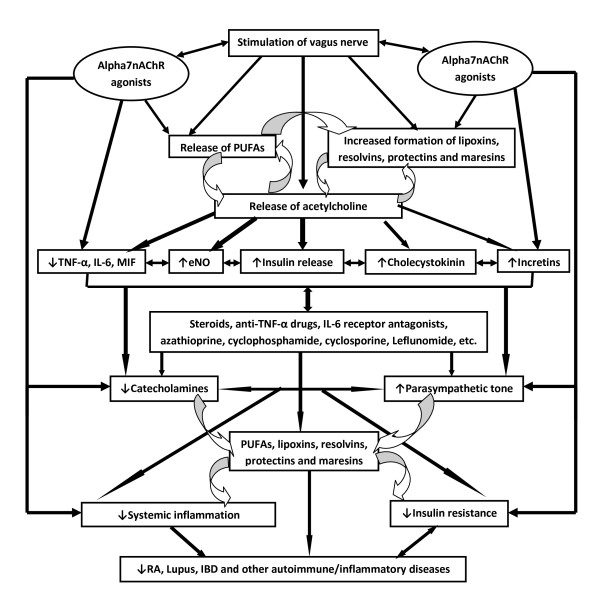
**Scheme showing the interaction among VNS, cytokines and PUFAs and their anti-inflammatory metabolites lipoxins, resolvins, protectins and maresins and their role in RA, lupus and IBD**.

It is evident from the preceding discussion that vagus nerve stimulation suppresses inflammation, alpha7nAChR agonists have the ability to ameliorate rheumatoid arthritis, especially in animal models of arthritis whereas arthritis could be exacerbated by vagotomy and suppressed by oral nicotine administration; oral nicotine and/or alpha7nAChR agonists inhibit bone degradation and reduced TNF-α expression in synovial tissue and reduced synovial inflammation (see Figure 1). Thus, vagal tone plays an important role in the pathobiology of inflammation and RA and possibly, other related conditions. Hence, it is proposed that in subjects with RA, lupus and other autoimmune inflammatory diseases the vagal tone is subnormal and sympathetic tone is high. Acetylcholine, the principal vagal neurotransmitter, suppresses inflammation by inhibiting proinflammatory cytokine release through a mechanism that requires alpha7nAChR [6,36-42], while catecholamines have proinflammatory actions [43,44]. Insulin resistance seen in RA, lupus and other autoimmune diseases could also be attributed to reduced vagal tone since, vagus has a regulatory role in insulin secretion and insulin resistance produced by bilateral cervical vagotomy can be reversed by acetylcholine [45-47]. Furthermore, acetylcholine and VNS ameliorate insulin resistance, in part, by enhancing the release of cholecystokinin and incretins [48].

As discussed above, VNS and PUFAs seem to interact with each other such that they potentiate each other's actions. Both VNS and PUFAs (and their products lipoxins, resolvins, protectins, maresins and PGI_2_) have anti-inflammatory actions, reduce insulin resistance, and are useful in the prevention and management of cardiovascular diseases and cardiac arrhythmias [7,8,11,26-28,34,36,45-50]. It is likely that in patients with RA, lupus and other autoimmune diseases and inflammatory conditions such as inflammatory bowel diseases, vagal tone will be subnormal and sympathetic tone is high; synovial fibroblasts, infiltrating (into the involved joints) macrophages, T cells and leukocytes may show reduced number or defective expression of alpha7nAChR and their content of PUFAs (especially AA, EPA and DHA) and ability to form adequate amounts of lipoxins, resolvins, protectins and maresins will be defective; and even the plasma and synovial fluid content of PUFAs and concentrations of lipoxins, resolvins, protectins and maresins are likely to be low. It is also proposed that there could be a defect in the synthesis (or inadequate formation) of lipoxins, resolvins, protectins and maresins from PUFAs and in response to alpha7nAChR and VNS in those who respond poorly to treatment and who have aggressive RA and lupus autoimmune diseases and inflammatory conditions such as inflammatory bowel diseases.

## Testing the hypothesis

It is proposed that reduced vagal tone triggers increased production of IL-6, TNF-α, MIF and HMGB1 by peripheral leukocytes, monocytes and macrophages; leads to an increase in sympathetic activity (due to the absence of negative feed-back control from the parasympathetic system), and consequent inflammation due to a decrease in the production of anti-inflammatory acetylcholine that ultimately results in systemic inflammation seen in RA and lupus. Hence, it is expected that an increase in plasma catecholamines and decrease in acetylcholine (and also in the synovial cells, peripheral leukocytes, macrophages and T cells) occurs in these patients. Peripheral leukocytes, macrophages and T cells and synovial cells obtained from the involved joint can be used for measuring the concentrations of catecholamines and acetylcholine in these subjects since they contain the complete intracellular machinery for the generation, release and inactivation of these molecules. Thus, measurement of catecholamines and acetylcholine in the plasma and peripheral leukocytes and vagal tone by HRV could be used as markers of inflammation and to predict, prognosticate and gauge response to treatment in patients with RA and lupus. In addition, plasma and tissue (especially of peripheral leukocytes, macrophages and T cells and synovial cells) concentrations of AA, EPA and DHA and their products lipoxins, resolvins, protectins and maresins are likely to be low in patients with RA and lupus. A negative correlation could exist between plasma, synovial, and leukocyte, macrophage and T cell content of acetylcholine, AA, EPA, DHA, lipoxins, resolvins, protectins and maresins and the severity of RA and lupus. It is proposed that VNS enhances the formation of lipoxins, resolvins, protectins and maresins from their precursor PUFAs. This could be verified by studying the effect of alpha7nAChR agonists on the synthesis and release of lipoxins, resolvins, protectins and maresins from PUFAs by leukocytes, macrophages, T cells, synovial cells and other cells in vitro. Similarly, in experimental animals and humans the ability of VNS to augment the formation of lipoxins, resolvins, maresins and protectins in various tissues and enhance their levels in the plasma could be assessed.

## Conclusions

The proposal that vagal tone is decreased, sympathetic tone is enhanced and increased production of IL-6, TNF-α, MIF and HMGB1 and decrease in plasma and tissue concentrations of AA, EPA and DHA and their products lipoxins, resolvins, protectins and maresins occurs in RA and lupus has important therapeutic implications. If true, it implies that methods designed to block α_2 _-adrenoreceptors [(since blocking these receptor inhibits inflammation injury due to catecholamines [43]], stimulation of the vagus nerve [8] and the nicotinic acetylcholine receptor α7 subunit [6] and supplementation of AA, EPA and DHA and administration of lipoxins, resolvins, protectins and maresins or their stable synthetic analogues are expected to be of significant benefit in the prevention and management of RA, lupus and inflammatory bowel diseases (Figure 1).

In an animal model wherein colitis was induced by intracolonic instillation of dinitrobenzene sulfonic acid, VNS (left cervical) reduced the degree of body weight loss, inflammatory markers as observed by histological score and myeloperoxidase quantification and an improvement of the multivariate index of colitis was noted. These data lend further support for an anti-inflammatory role of VNS in rats with colitis and provide potential therapeutic applications for patients with inflammatory bowel disease [51].

Similar to the beneficial action of VNS in inflammatory bowel disease, lipoxins (derived from PUFAs) are also useful in resolving inflammatory bowel disease. LXs are produced by gut mucosa. Colonic mucosa from patients with ulcerative colitis produced significantly lower (12-fold) amounts of LXA_4 _compared with control [52], and mice chronically treated with a selective 15-lipoxygenase inhibitor experienced significantly worse intestinal function during experimental colitis, relative to untreated mice. On the other hand, an orally effective and a beta-oxidation-resistant 3-oxa- aspirin-triggered 15-epi-lipoxin (ATL) analog (ZK-192) markedly attenuated trinitrobenzenesulphonate (TNBS)-induced colitis. ZK-192 decreased mucosal mRNA levels of inflammatory mediators: inducible nitric oxide synthase, cyclooxygenase 2, and macrophage inflammatory protein 2, also decreased mucosal mRNA and protein levels of T helper 1 effector cytokines: TNF-α, IL-2, and IFN-γ and reduced the systemic levels of these cytokines. ZK-192 also dramatically attenuated CD3/CD28-mediated costimulation of T helper 1 effector cytokine release in lamina propria mononuclear cells [53]. Thus, both VNS and LXs have similar anti-inflammatory actions in RA, lupus and inflammatory bowel disease.

Vagal nerve stimulation (VNS) is already in clinical use as an adjunctive treatment for certain types of intractable epilepsy and major depression [54,55]. VNS uses an implanted stimulator that sends electric impulses to the left vagus nerve in the neck via a lead wire implanted under the skin. The advantage of VNS is that it can be done as an out-patient procedure. It is possible to pharmacologically target the alpha7nAChR dependent control of cytokine release in RA and lupus patients with suppressed vagus nerve activity. As alpha7nAChR agonists ameliorate the clinical course of collagen induced arthritis in animals, it may be possible in the future to explore whether alpha7nAChR agonists can improve clinical activity in RA and lupus patients. In this context, it will be interesting to study the effect of steroids, anti-TNF-α drugs, immunosuppressive drugs such as azathioprine, cyclophosphamide, cyclosporine, etc., on their ability to modulate alpha7nAChR and vagal tone and the formation of lipoxins, resolvins, protectins and maresins from various PUFAs and are thus, able to bring about some of their beneficial actions in RA and lupus. It is likely that in future in addition to the currently available treatment regimens one could employ VNS and alpha7nAChR agonists, stable and synthetic analogues of lipoxins, resolvins, protectins and maresins in the management of RA, lupus, inflammatory bowel diseases and other inflammatory and autoimmune disorders.

## Competing interests

The authors declare that they have no competing interests.
